# Diagnostic challenges in minimal deviation adenocarcinoma of the uterine cervix: A report of two cases and review of the literature

**DOI:** 10.3892/mco.2013.144

**Published:** 2013-07-08

**Authors:** FEIFEI GUO, YALI HU, XIAOFENG XU, RONG LI, TONG RU, JINGMEI WANG, HUAIJUN ZHOU

**Affiliations:** 1 Department of Obstetrics and Gynecology, Nanjing Drum Tower Hospital, The Affiliated Hospital of Nanjing University Medical School, Nanjing, Jiangsu 210008, P.R. China; 2 Department of Pathology, Nanjing Drum Tower Hospital, The Affiliated Hospital of Nanjing University Medical School, Nanjing, Jiangsu 210008, P.R. China

**Keywords:** minimal deviation adenocarcinoma, uterine cervix, case report, literature

## Abstract

Minimal deviation adenocarcinoma (MDA) of the uterine cervix, otherwise known as adenoma malignum, is a rare variant of cervical adenocarcinoma, which represents a diagnostic challenge in the field of gynecologic oncology, due to its benign-resembling histological characteristics. To achieve a better understanding of this disease, we present two cases of MDA: one case presented with profuse watery discharge and cervical enlargement, accompanied by retention cysts and hardening; the other presented with a history of myoma cervicis uteri. Both patients underwent total abdominal hysterectomy, bilateral salpingo-oophorectomy and pelvic lymphadenectomy and our follow-up indicated that the patients were still free of any disease. Subsequently, a literature review was performed and the results demonstrated that early diagnosis, clinical stage and surgical protocols are the main factors affecting the prognosis of MDA. Close follow-up of the cases may provide more information regarding this disease and the efficacy of the available therapeutic methods.

## Introduction

Minimal deviation adenocarcinoma of the uterine cervix (MDA), otherwise known as adenoma malignum, is a rare variant of cervical adenocarcinoma, which represents a diagnostic challenge in the field of gynecologic oncology. It is a rare neoplasm with an incidence of 1–3% and was first designated as ‘malignant adenoma of the cervix’ by Gusserow (1). However, Silverberg and Hurt (2) proposed the term ‘minimal deviation adenocarcinoma’ for this tumor due to its deceptively benign microscopic appearance. Since that time, only a few cases of MDA have been reported in the English literature.

In this study, we present two cases of MDA, in order to demonstrate the characteristics, diagnostic and therapeutic strategies that distinguish it from ordinary endometrioid adenocarcinoma.

## Case reports

### Case 1

A 45-year-old, multiparous woman (gravida 3, para 1, G3P1) presented with a 5-year history of large amounts of vaginal discharge. The ThinPrep cytology test revealed moderate inflammation. Several transvaginal ultrasonography scans revealed an edematous cervix and multiple cysts with a honeycomb appearance (Fig. 2B). The inner cervical wall was not smooth and the tumor marker levels were within the normal range. Following cervical conization for the cervical cysts, the biopsies revealed chronic cervical inflammation with the presence of retention cysts and squamous metaplasia in the fundic portion of the cervix. Subsequently, the patient underwent laparoscopic cystectomy and biopsy, hysteroscopy, fractional curettage and cervical biopsy. The histopathological examination revealed chronic inflammation of the cervical mucosa. However, the vaginal discharge did not subside. The patient then underwent a pelvic magnetic resonance imaging (MRI) examination, which revealed multiple cervical cysts and hydrops in the pelvic cavity. Medically the patient was in good condition and her history only revealed an appendectomy performed in 1983. Following admission to our department, the gynecologic examination showed large amounts of vaginal discharge and cervical hypertrophy, with no other abnormal findings. The patient underwent total abdominal hysterectomy and the fast-frozen cervical biopsy revealed the presence of adenocarcinoma; thus, bilateral salpingo-oophorectomy and pelvic lymphadenectomy was also performed.

**Figure 1:**
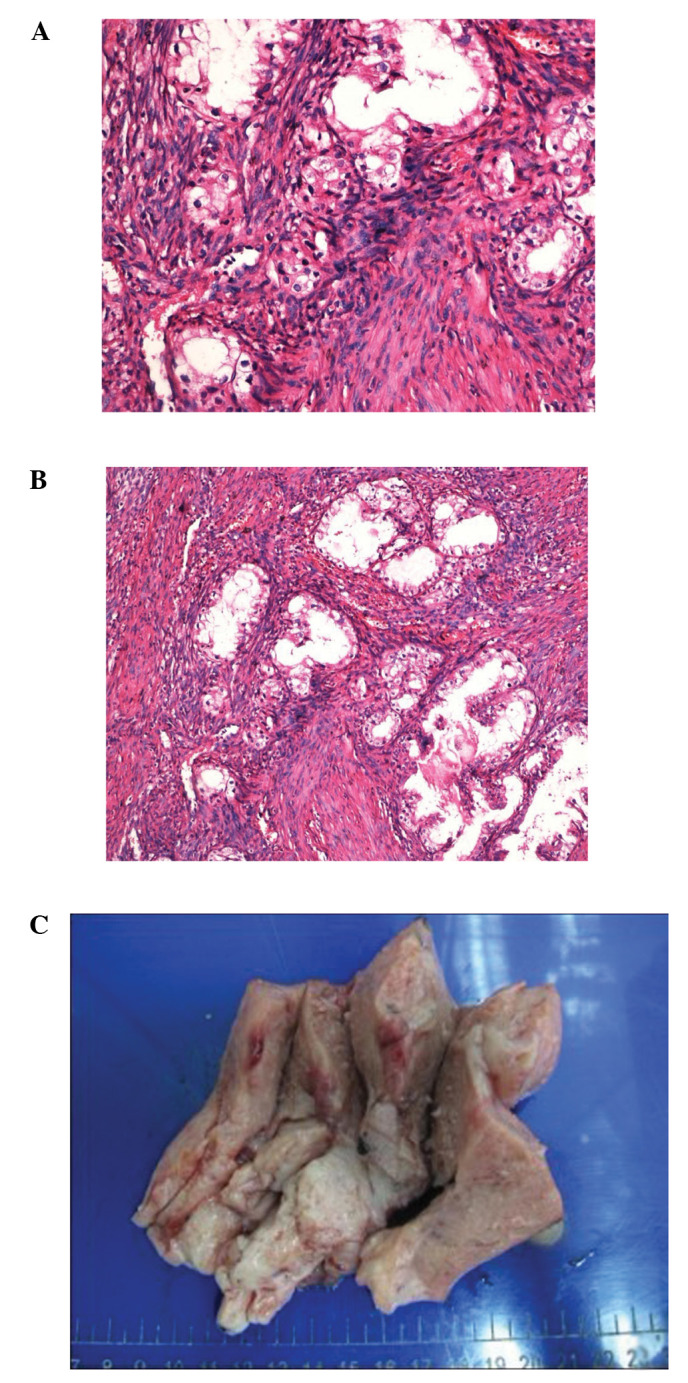
(A and B) Histopathological characteristics of minimal deviation adenocarcinoma (MDA) for the first case. Microscopically, the lesion was characterized by mucinous glands which resembled normal endocervical glands, exhibiting, however, distinct nuclear anaplasia or evidence of stromal invasion. Hematoxylin and eosin (H&E) staining, magnification (A) ×100 and (B) ×200. (C) Appearance of the MDA tumor specimen doused in formaldehyde. The cervix was thickened, measuring 4 cm in diameter, exhibiting multiple retention cysts and hardening. No other gross abnormalities were observed. The ovaries and fallopian tubes had a normal appearance and the histological evaluation revealed no abnormalities.

**Figure 2:**
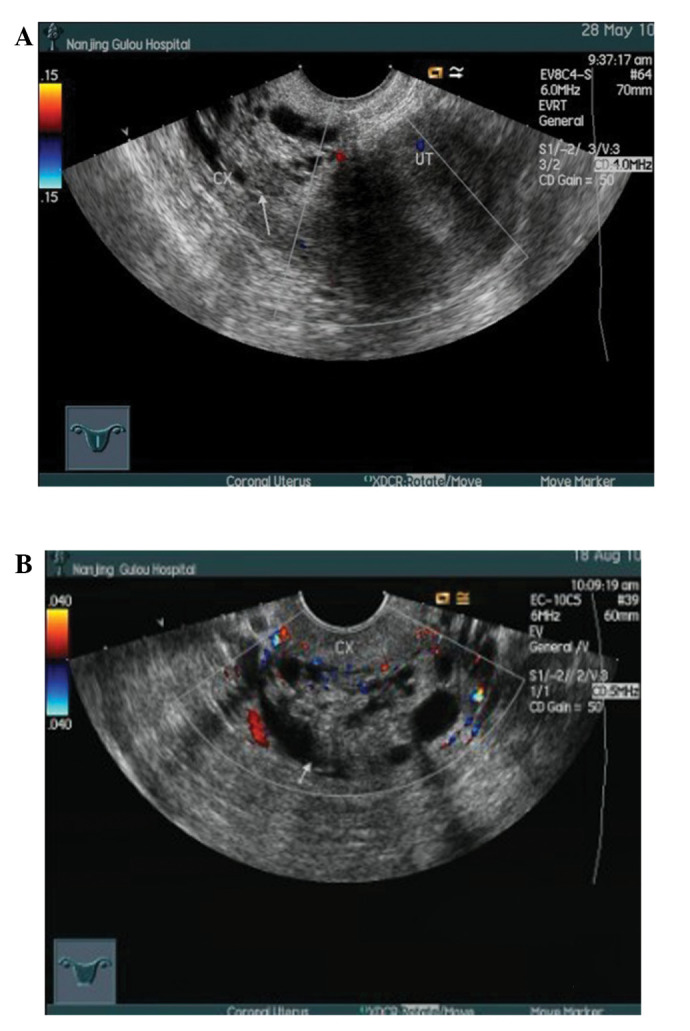
Transvaginal ultrasonographic characteristics of minimal deviation adenocarcinoma of the uterine cervix for the first case. (A) Enlarged uterus (5.3 × 6.3 × 5.1 cm), with a honeycomb appearance. (B) Detailed information of the cervix.

Grossly, the cervix was thickened and hard with multiple retention cysts, with no other abnormal macroscopic findings (Fig. 1C). The microscopic examination revealed cervical mucilaginous glands that were irregular in size and shape with increased apophysis, part of the glands were surrounded by a loose edematous or desmoplastic stromal response, the glands typically exhibited deep invasion of the cervical wall and were adjacent to the cervical adventitia. The glandular epithelial cells exhibited foci of heteromorphism. The parametrium and the pelvic lymph nodes showed no evidence of malignancy. The tumor was staged as Ib2 MDA according to the FIGO classification. Subsequently, cervical and pelvic radiotherapy was performed. At the last follow-up the patient was disease-free.

### Case 2

A 41-year-old, multiparous woman (G6P1) underwent myomectomy for a cervical hysteromyoma in 2011 and pathological examination of the hysteromyoma revealed an MDA. The patient was medically fit and in good overall condition. Her medical history revealed myomectomy and oophoritic cystectomy (10 years ago). The gynecologic examination showed cervical moderate inflammation, with no other abnormal findings. The patient subsequently underwent total abdominal hysterectomy, bilateral salpingo-oophorectomy and pelvic lymphadenectomy.

The gross uterine appearance was normal, apart from an enlarged corpus. The microscopic examination revealed cervical chronic inflammation with retention cysts and squamous metaplasia, adenomyosis and chronic salpingitis. The pelvic lymph nodes exhibited reactive hyperplasia, with no other abnormalities. The tumor was stage Ib and there were no high risk factors for the patient; therefore, adjunctive therapy was not administered. At the last follow-up the patient exhibited no evidence of tumor recurrence.

## Discussion

To gain more insight into MDA, we performed a review of the literature, during which 60 cases were identified (Table I). In almost half of these cases, vaginal bleeding and discharge was the predominant symptom and pathological examination was used to confirm the diagnosis. Surgical resection was the first choice for the treatment of MDA.

The origin of MDA remains unclear. Previous studies revealed that the tumor is likely unrelated to human papillomavirus infection, which distinguishes it from common cervical cancers (14). Certain studies demonstrated a close association between MDA and gastric metaplasia. McGowan *et al*(15), suggested that the existence of Peutz-Jeghers syndrome or ovarian tumors may contribute to the progress of MDA, although no definitive conclusion on this association was established in our cases. The symptoms and signs of MDA are not different from those of common cervical adenocarcinoma. In our first case, the patient presented with profuse watery discharge and enlarged cervix with retention cysts. The cytological examination, punch biopsy and cervical conization failed to confirm a diagnosis of MDA. Therefore, in patients with cervical hypertrophy presenting with vaginal discharge or irregular bleeding, MDA should be considered following the elimination of other possible causes (such as carcinoma tubae)and appropriate investigations should be conducted, leading to a definitive diagnosis. The diagnosis of MDA is based on histopathology. Previous studies demonstrated that the cytological examination of the cervix as a diagnostic method for MDA is not sufficient.

**Figure 3:**
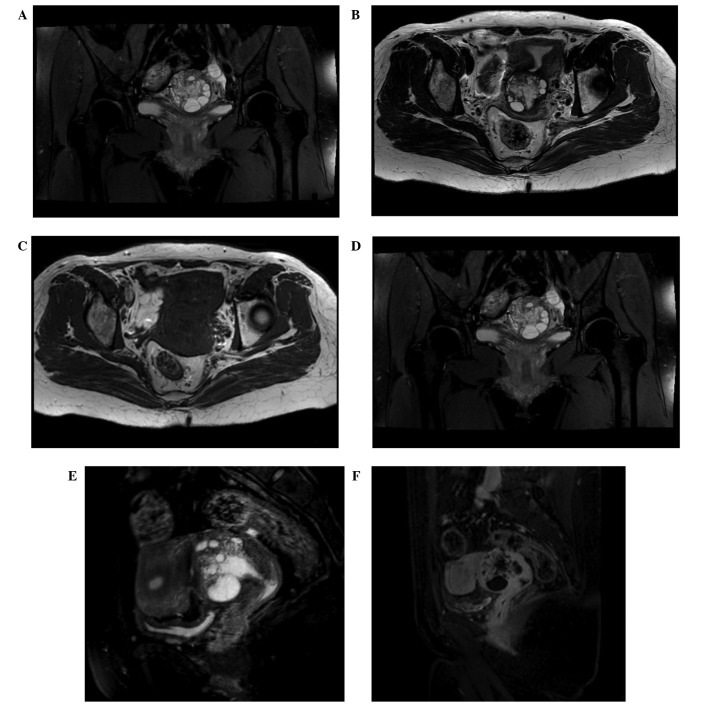
Magnetic resonance imaging (MRI) appearance of minimal deviation adenocarcinoma (MDA). (A) Proton-density-weighted-spectral adiabatic inversion recovery (PDW-SPAIR), coronal plane; (B) T2-weighted image (T2WI); (C) T1-weighted image (T1WI); (D) T1WI-transverse section; (E) T2WI-SPAIR, sagittal plane; (F) T1WI, sagittal plane.

However, biopsy of the cervix and the cervical canal (depth >5 mm) and cervical conization contribute to the definitive diagnosis of MDA (16). Diagnosis using imaging techniques, such as MRI and ultrasonography, is often difficult due to the benign appearance of this tumor; however, they play an important role in evaluating the dissemination of MDA (17). T2-weighted MRI, in particular, shows the characteristics of MDA in detail and exhibits a reliable correlation with histological findings (18). In our first case, the T2-weighted MRI revealed a multicystic lesion and fluid accumulation in the endometrial cavity (Fig. 3B). MDA exhibits a diffusely infiltrative growth pattern and its differentiation from normal cervical glands histologically is challenging (19). However, MDA is histologically characterised by the haphazardous arrangement of endocervical glands and their deep penetration into the cervical wall, with only minor cytological atypia. Immunohistochemistry usually serves as an auxiliary examination of morphology to distinguish MDA from other cervical diseases. Previous studies revealed that carcinoembryonic antigen, Ki67, alcian blue-periodic acid-Schiff staining and p53 may play important roles in the disease aetiology (20). Currently, surgery remains the optimal treatment choice for MDA. The modus operandi for the patients without a definitive diagnosis should be the same as that for ordinary adenocarcinoma. However, postoperative adjunctive therapy may be required for patients with MDA, as the disease is usually diagnosed at a later stage. From the prognostic point of view, a firm conclusion cannot be reached, due to the limited number of reported MDA cases and the limited clinical follow-up. In our two cases, opportune diagnosis allowed application of the appropriate treatment, similar to an ordinary well-differentiated adenocarcinoma. Close follow-up of our two cases was planned in order to obtain more information about the disease and the efficacy of the available therapeutic methods.

In conclusion, early diagnosis followed by appropriate ancillary evaluation and treatment have been a challenge for gynecologists. Close follow-up of established cases is essential in gaining more information regarding the disease and the efficacy of the available therapeutic methods.

## Figures and Tables

**Table I:** Data of 60 cases of minimal deviation carcinoma of cervix.

Study (n)	Age (years)	Presenting symptom (n)	Treatment (n)	Stage (n)	Cytology (n)	Pathology (n)	IHC (n)	Prognosis (n)	Refs.
Chang *et al*(5)	38–59	Atypical vaginal discharge (3)	Rad (5)	Ib (2)	Adenoma malignum (2)	MDA (5)		Succumbed to the disease (3)	(3)
			Radical hysterectomy with pelvic node dis (3)	Iib (1)	Ordinary adenocacinoma (1)		CEA+, p53+ (2)		
				IIIb (1)			Unknown (3)	NED (2)	
			AH and BSO (1)	IV (1)	No malignancy (2)				
Simionescu *et al*(1)	32	Atypical vaginal discharge and bleeding	Cx Bx	Unknown	Normal	MDA	CEA+, CA125+, Ki67+	Unknown	(4)
Steeper *et al*(4)	38–74	Vaginal bleeding (3)	Radical hysterectomy (3)	Ia (1)	Unknown	MDA	CEA+	Succumbed to the disease (3)	(5)
		Vaginal discharge (1)	Rad (1)	Ib (2)					
				Iib (1)				NED (1)	
Du *et al*(1)	27	Vaginal discharge and bleeding	Radical hysterectomy, pelvic node dis	Unknown	Not performed	MDA	CEA+, p53+, Ki67 (10%)	NED	(6)
Yang *et al*(14)	31–63	Vaginal bleeding (9)	Unknown	I (4)	Adenoma malignum (1)	MDA	CEA+ (12)	Unknown	(7)
		Vaginal discharge (12)		II (7)	Normal (9)		Ki67+ (11)		
				III (2)	Unknown (4)		p53+ (8)		
				IV (1)					
Zhang *et al*(9)	36–50	Unknown	Unknown	Unknown	Unknown	Unknown	CEA+ (6); α-SMA+ (8)	Unknown	(8)
							Ki67+ (9)		
Jiang *et al*(1)	61	Leucorrhea with blood streak, menopause	AH, BSO	Unknown	No malignancy	MDA	Not performed	NED	(9)
Abiko *et al*(1)	56	Vaginal discharge	AH, BSO, pelvic and para-aortic node dis	Unknown	Not performed	MDA	CEA+; CA19-9+	Unknown	(10)
							MUC6+; HIK1083+		
Odashiro *et al*(3)	30–45	Blood-tinted vaginal discharge	Cx and pelvic rad, followed by radical hysterectomy	Unknown	Adenocarcinoma *in situ*(1) vs. well-differentiated adenocarcinoma (2)	MDA diagnosed by Cx Bx	Vimentin+	Succumbed to metastatic disease (1)	(11)
								NED (2)	
Chen (8)	26–68	Vaginal discharge (7)	AH, BSO, pelvic node dis	Unknown	Unknown	MDA diagnosed by Cx Bx	CEA+; CK7+; CK19+; CA19-9+; Vimentin+; SMA+	Unknown	(12)
		Vaginal bleeding (6)							
		Contact bleeding (2)							
		Menolipsis (1)							
Kaminski *et al*(13)	31–76	Vaginal bleeding (7)	AH (4)	Ib (12)	Not performed (7)	Endometrioid (7)	Unknown	NED, ≥9 years (5)	(13)
		Vaginal discharge (3)	VH (1)	Iib (1)	Class 1 (4)	Mucinous (5)		Succumbed to the disease (6) Lost (2)	
		Cervical stenosis with associated pyometra (1)	D&C, cone, WH, pelvic node dis (2)		Class 3 (2)	Clear-cell (1)			
		Abnormal Pap smear and bleeding (1)	D&C, cone, WH, pelvic node dis and postoperative rad (2)						
		Cervical neoplasm (2)							
			D&C, cone, WH, pelvic node dis and VH (1)						
			Cx Bx, AH (1)						
			D&C, cone (1)						
			AH, BSO (1)						

A literature search was conducted in MEDLINE, China Periodical Full-Text, Chinese Science and Technology Periodical and WanFang Information Resources System databases, using the terms: ‘minimal deviation carcinoma and uterine cervix’ in English or Chinese. References of relevant studies were manually searched. Studies reporting unclear information regarding diagnosis of pathology were excluded. Rad, radiation; MDA, minimal deviation adenocarcinoma; NED, no evidence of disease; dis, dissection; AH, abdominal hysterectomy; BSO, bilateral salpingo-oophorectomy; Cx, cervix; Bx, biopsy; D&C, dilatation and curettage; Cone, conization; VH, vaginal hysterectomy; WH, Wertheim hysterectomy; CEA, carcinoembryonic antigen; SMA, smooth muscle actin; CK, cytokeratin; CA 19-9, carbohydrate antigen 19-9; IHC, immunohistochemistry; n, patient number.
